# Harvard Medical School

**Published:** 1880-06

**Authors:** 


### HARVARD MEDICAL SCHOOL.

The prosperity of the Medical School continues and increases In 1878-'79 the number of students increased ten per cent., and

33

the excess of receipts over expenditures was $9,540.07, although each of the clinical instructors received an honorarium, which was a new charge upon the school. The number of students who possess literary or scientific degrees has doubled in ten years, and now amounts to 48 per cent. of the whole number.—*President Ellicott's Report.*

